# A delayed gastric antral vascular ectasia: A case report and literature review

**DOI:** 10.1097/MD.0000000000040831

**Published:** 2024-12-27

**Authors:** Zheke Fang, Jiajie Zhu, Zheng Fang, Qiang Hu, Liangjun Yang

**Affiliations:** a Department of Rehabilitation, Tongde Hospital of Zhejiang Province, Hangzhou, Zhejiang Province, China; b Zhejiang Academy of Traditional Chinese Medicine, Hangzhou, Zhejiang Province, China; c Department of Gastroenterology, Tongde Hospital of Zhejiang Province, Hangzhou, Zhejiang Province, China; d Department of General Surgery, Tongde Hospital of Zhejiang Province, Hangzhou, Zhejiang Province, China.

**Keywords:** argon plasma coagulation, endoscopic band ligation, endoscopy, gastric antral vascular ectasia, watermelon stomach

## Abstract

**Rationale:**

Gastric antral vascular ectasia (GAVE) is a rare cause of gastrointestinal bleeding. It presents challenges in both diagnosis and treatment.

**Patient concern:**

We reported a female patient who was initially misdiagnosed with hemorrhage of the digestive tract. She was treated with medication for 4 months, but her hemoglobin levels still declined without blood transfusion.

**Diagnosis:**

Her diagnosis was GAVE after an endoscopic examination.

**Interventions:**

She was treated with an endoscopic argon plasma coagulation treatment, an endoscopic band ligation treatment, medication, and traditional Chinese medicine.

**Outcomes:**

This case was followed up for 6 months and her hemoglobin levels were above 9 g/dL.

**Lessons:**

A combination treatment of endoscopic and medication treatment will be a good choice of GAVE. More comprehensive understanding of GAVE will be build up with the developments of new technologies and methods.

## 1. Introduction

Gastric antral vascular ectasia (GAVE) is a rare form of occult gastrointestinal bleeding, and accounts for 4% of non-variceal upper gastrointestinal bleeding.^[1]^ GAVE was first reported by Rider et al in 1953.^[2]^ And it is gradually known by Jabbari et al who described 1 characteristic appearance of GAVE as “stripes of a watermelon” in 1984.^[3]^ The exact cause of its onset remains unclear, but GAVE is often associated with systemic sclerosis, chronic renal failure, cirrhosis and bone marrow transplantation.^[4–6]^ And it presents with symptoms of iron deficiency anemia, bloody stool, and fatigue.

We present a patient who was initially misdiagnosed with hemorrhage of the digestive tract at other hospitals. Her final diagnosis was GAVE after an endoscopic examination. Then, she was treated with an argon plasma coagulation (APC) treatment, an endoscopic band ligation (EBL) treatment, and medication. Her process of GAVE reveals some issues and reflection.

## 2. Case presentation

In this case, a 78-year-old female, whose chief complaints were melena and persistent fatigue over 4 months, was initially diagnosed with hemorrhage of the digestive tract, cirrhosis, severe anemia, and hypoproteinemia at other hospitals. She had received pharmacotherapy, including octreotide, for 4 months, but her hemoglobin (Hb) level still declined without blood transfusion. However, none of these hospitals performed an endoscopic examination for her.

On July 12th, the weak patient was sent to our hospital. Upon admission, we inquired in detail about her medical history and learned that she had hypertension for 8 years, splenomegaly for 5 years, Type 2 diabetes for 3 years, coronary heart disease for 3 months, and urinary retention for 5 months. A quick blood examination revealed her Hb level was 6 g/dL, white blood cell count was 2.1 × 10^9^/L, red blood cell count was 1.89 × 10^12^/L, and platelet count was 56 × 10^9^/L. Her fecal occult blood test was 3+. And her vital signs were within normal range. We conducted an abdominal CT which revealed cirrhosis, splenomegaly, and gastric fundus varices. This easily yields results similar to other hospitals – cirrhosis and hemorrhage of the digestive tract.

**Figure 1.**
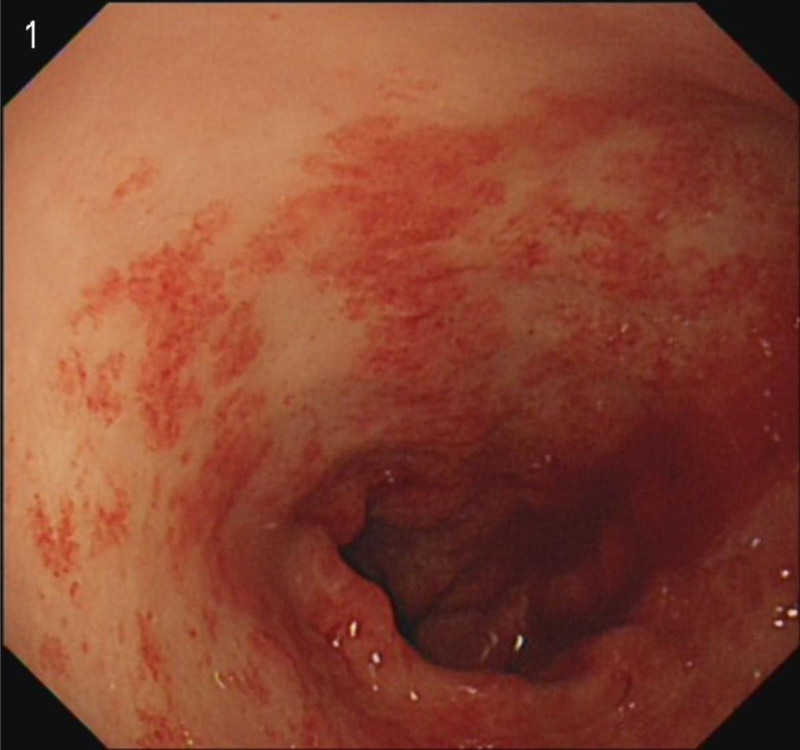
Initial appearance.

**Figure 2.**
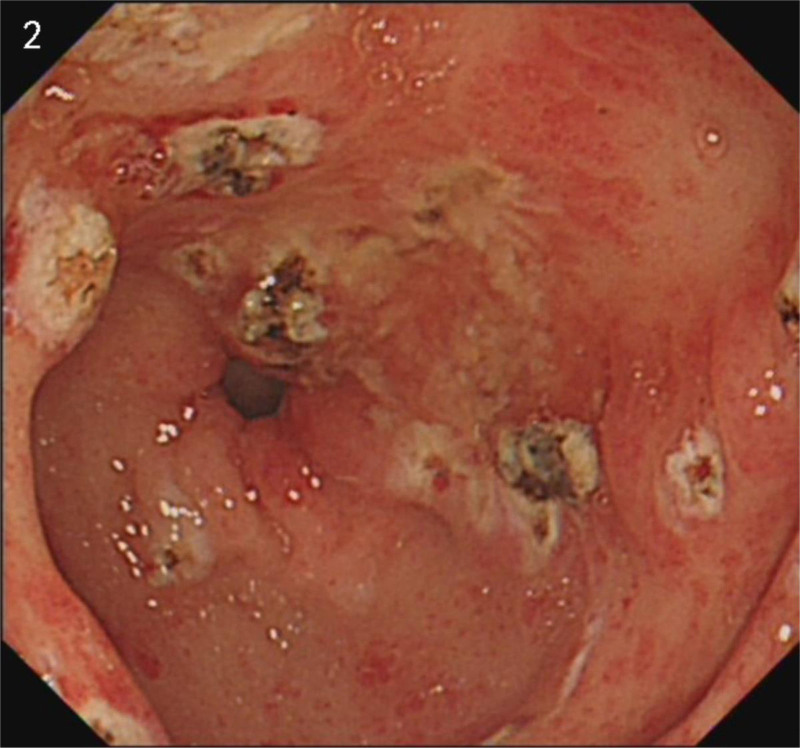
After an APC treatment. APC = argon plasma coagulation.

**Figure 3.**
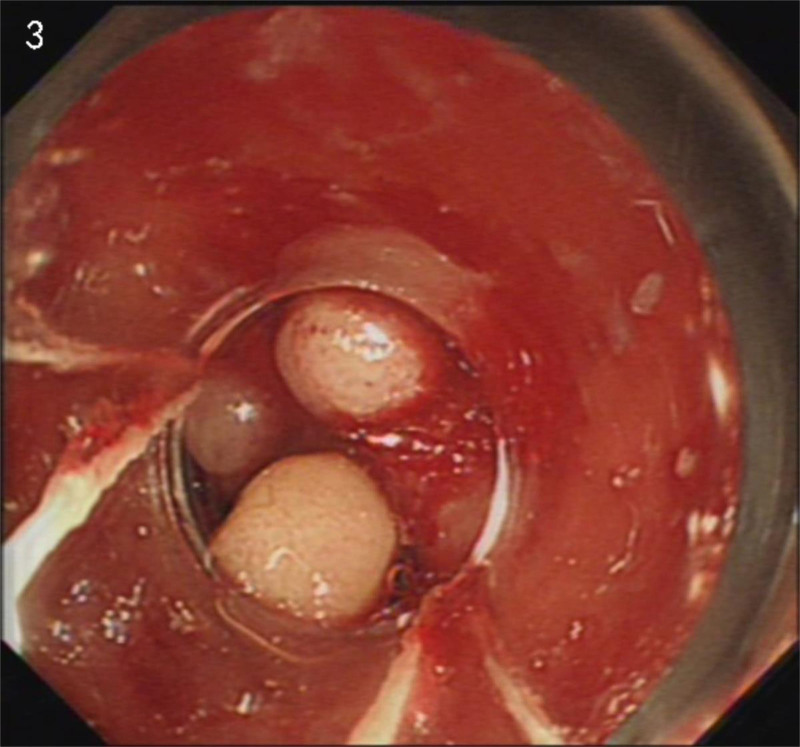
After an EBL treatment. EBL = endoscopic band ligation.

Considering the ineffectiveness of previous treatments at other hospitals, we persisted with an endoscopic examination. It wasn’t until July 29th that the patient agreed to do an examination. The endoscopic examination revealed a typical “watermelon” appearance (Fig. 1). Combined with CT results, we reached the conclusion of GAVE at last. Four days later, the patient underwent APC treatment (Fig. 2). At the same time, we used thalidomide 25 mg twice a day to assist with hemostasis. The patient’s Hb level initially increased but subsequently decreased on August 13th. Based on our experience, we recommended an EBL as an alternative treatment (Fig. 3). And we increased the dosage of thalidomide to 50 mg twice a day. The patient was discharged on August 19th as her Hb level reached a higher level. On August 29th, the patient reported her Hb level was 9.0 g/dL. One week later, the patient developed a generalized rash, which may be a side effect of thalidomide. Therefore, we advised the patient to stop the use of thalidomide, and the rash disappeared soon. To consolidate the treatment effect, we used traditional Chinese medicine. The self-formulated prescription was as follows: Ku Shen 15 g, Tu Fu Ling 30 g, Dan Shen 30 g, Qian Cao Tan 30 g, Ban Zhi Lian 30 g, White Flower Snake Tongue Grass 30 g, Astragalus 100 g, San Qi 12 g, Hai Piao Xiao 15 g, Fu Chao Zhi Shi 10 g, Chao Zi Su Zi 10 g, Cu Xiang Fu 10 g, Sha Ren 6 g, Tai Zi Shen 30 g, Bai Zhu 10 g, Dang Shen 30 g, Dang Gui 10 g, and Gan Jiang 7 g. Over the next 6 months, we followed up on her condition. Her Hb levels remained above 9.0 g/dL, and she did not undergo any further endoscopic treatment or blood transfusion.

## 3. Discussion

### 3.1. The recognition of GAVE is inadequate

Firstly, the incidence rate of GAVE is low and literatures of GAVE is limited. We have searched English databases, including PubMed, which presented 583 literature till July 31st, 2024. Most of them are case reports and only 3 are randomized controlled trials (RCTs).^[7–9]^ And we also find 107 articles in Chinese databases, including CNKI, CQVIP, and WANFANG DATA. Limited articles cannot support sufficient guidance on clinical practice. This may be the main reason of a lack of awareness among a plenty of clinical physicians, which will result in diagnostic delays or misdiagnoses. Besides, only a few studies have conducted long-term follow-ups.^[10–12]^ Follow-up is essential for evaluating the efficacy. And, most studies focus on single treatment regimens, which may not reflect the clinical reality.

Secondly, the theoretical system is in obscure. Several theories have been proposed to explain GAVE’s development. One theory focuses on hormonal changes, including alterations in gastrin, prostaglandin E2, vasoactive intestinal polypeptide, and serotonin levels.^[4,13,14]^ Another theory highlights the role of increased mechanical stress on the antrum, resulting in fibromuscular hyperplasia and chronic compression of submucosal vessels.^[15]^ And, 1 recent research has indicated a potential correlation between GAVE and Vitamin D.^[16]^ Whether GAVE is a disease which is acquired lesions or congenital vascular abnormalities, and whether it is a primary disorder or a secondary manifestation, are still unclear.

The clinical pressure faced by gastroenterologists cannot be ignored. In this case, the patient with poor health condition faces risks of secondary bleeding during endoscopic examinations. Thus, many clinicians, upon observing vascular dilation post-cirrhosis, fear the risks associated with endoscopic examination and opt solely for pharmaceutical treatment. Although a cautious approach may seem prudent, it is actually irresponsible towards patients and hampers a deeper understanding of GAVE.

### 3.2. The diagnosis of GAVE remains unclear

Histology is not a “gold standard” in diagnosing GAVE. Pathological features of GAVE comprise 3 main parts: (1) spindle cell proliferation presenting; (2) with either ectasia, fibrin thrombi, or a combination of both ectasia and fibrin thrombi; (3) fibrohyalinosis.^[17,18]^ However, vascular ectasia, fibrohyalinosis, and fibrin thrombi can also be present in gastric hyperplastic polyps and occasionally in gastritis. Therefore, biopsy should only be employed in situations where it is necessary to confirm a suspected diagnosis of GAVE.^[19]^

Due to the absence of definitive reliable characteristics, GAVE’s diagnosis primarily relies on gastrointestinal manifestations. Currently, at least 3 subtypes of GAVE exist^[20]^: (1) striped pattern (watermelon pattern)^[3]^; (2) punctate pattern (honeycomb pattern)^[21]^; and (3) nodular pattern.^[22]^ It has been observed that the linear subtype is more frequently observed in non-cirrhotic female patients, while the diffuse subtype tends to occur in male cirrhotic patients.^[23]^ A more recently discovered subtype, nodular GAVE, is characterized by benign-appearing smooth nodules primarily located in the antrum and accounts for 30% of GAVE cases.^[20]^ In some cases, GAVE’s endoscopic appearance may present a similar pattern like portal hypertensive gastropathy or other diseases.^[18,24]^ Therefore, the diagnosis of GAVE requires a lot of experience.

We suggest solving the issue from 3 aspects: (1) establishing a comprehensive machine-comparing database. With advancements in artificial intelligence, a vast array of images can be gathered and analyzed to identify endoscopic characteristics and CT imaging of GAVE. Based on the 2 databases, we can compare endoscopic characteristics to the CT imaging. After understanding the correlation between the 2 databases, this is of significant help in diagnosing GAVE. This approach not only reduces human error in interpretation but also facilitates the creation of a disease database. (2) Enhancing the collection of blood indicators. Many doctors and patients are concerned about potential bleeding risks from endoscopic examinations. Thus, blood indicator assessments are vital. Westerhoff et al discovered that CD61 (a platelet marker) exhibited a 100% positive rate for GAVE, in contrast to a 60% positive rate for suspicious GAVE and an 18% positive rate for portal hypertensive gastropathy.^[25]^ The exploration of blood indicators holds great promise. (3) Enhancing diagnostic genetic testing. GAVE is usually found in elderly females (>70 years old), and only a few in children.^[26]^ Elliot et al discovered that 64% of GAVE patients were cirrhotic compared to 14% of controls. And the mean BMI, diabetes mellitus, and vascular disease were also higher than controls. Based on these, they speculated that GAVE is related to metabolic syndrome.^[27]^ Besides, researchers have found that the prevalence of GAVE with cirrhosis may be higher in Egypt compared to other regions.^[28]^ And in a study conducted in Ogun State, Nigeria, GAVE was found to contribute to 1.2% of 168 upper gastrointestinal bleeding patients.^[29]^ These may demonstrate a correlation in gene. By comparing the genes of GAVE to the healthy individuals, valuable insights can be gained. Studying the genes of GAVE across different ages, genders, and ethnicities can enhance our understanding.

### 3.3. Treating GAVE poses challenges

Currently, there are mainly 3 treatments: endoscopic treatment, medication therapy, and surgical treatment. Endoscopic treatments and surgical treatments aim to remove affected tissues, while medication therapies focus on reducing the accumulation of related factors.

Endoscopic treatments currently hold a prominent position. Endoscopic treatments include APC, EBL, neodymium:Yag (Nd-YAG) laser, radiofrequency ablation, heater probe, argon laser, sclerotherapy, and so on. According to the literature, the earliest common regimen was Nd-YAG laser,^[30]^ followed by APC. Additionally, EBL and RAF have gradually gained traction. Currently, the most widely used are APC and EBL and Nd-YAG laser is gradually abandoned for its side effects. In general, endoscopic treatments do solve the hemorrhage directly, but they usually require multiple sessions.^[31]^ And how to spare the overlying normal mucosa after endoscopic treatment remains a significant challenge.

APC has its notable advantages, such as safety, rapid hemostasis, minimal complications, and suitability for senior patients. Simple APC can only affect the mucosal layer and a part of pathological tissue. In other words, it means APC can only influence on a part of shallow tissues and will cause a high recurrence rate. And the high recurrence rate poses concerns on further risks and financial burdens. In order to solve this problem, various improved strategies have emerged. One strategy has combined APC with polidocanol injection, which has demonstrated better efficacy and a lower recurrence rate by influencing both the mucosal and submucosal layers.^[32]^ Another modified procedure, known as Hybird-APC, injects saline into the submucosa to create a safety cushion to ensure deeper treatment.^[30]^

EBL was first utilized in 2006 as a salvage therapy when APC was ineffective on GAVE.^[33]^ EBL can impact on the mucosa layer as well as the submucosa layer. Deeper treatment than APC shows better outcomes compared to simple APC in post-procedural blood transfusion, Hb increase, and fewer sessions.^[34]^ In a retrospective study by Sato et al, the recurrence rate of cirrhosis-associated GAVE treated with EBL was significantly lower compared to APC (8.3% vs 68.2%).^[35]^ A RCTs of EBL and APC revealed that EBL is more effective and comparable in safety.^[7]^ A RCTs showed that EBL is superior to APC in endoscopic eradication rates, recurrence of bleeding, and transfusion requirements.^[8]^ But, EBL has not been adopted worldwide, especially in underdeveloped regions.

Surgery is currently considered the only method to cure GAVE. However, the postoperative mortality rate can be 7.4%. Surgical options include Billroth I, Roux-en-Y, Billroth II, and total gastrectomy. Considering that GAVE patients are generally seniors and often do not have good surgical indications, surgery should be considered as a last resort in acute bleeding cases or when other treatment methods are ineffective.

Medication therapy has a longer history than endoscopy and surgery treatments in the management of GAVE. Medications primarily intervene in 3 aspects: (1) splanchnic vasoconstriction; (2) immunosuppression; (3) inhibition of angiogenesis. Octreotide, for example, exerts negative modulatory effects on vasoactive substances,^[36]^ reducing their stimulation to submucosal capillaries and alleviating bleeding. Cyclophosphamide and glucocorticoids can affect the immune system with different dosages.^[37]^ In terms of angiogenesis inhibition, thalidomide has shown effectiveness in liver cirrhosis patients with refractory GAVE.^[38]^ While most GAVE patients are seniors, the manageable side effects of thalidomide make it a viable option. As for other medicines like calcitonin, bevacizumab and tranexamic acid, their effect on GAVE has not been sufficiently confirmed. However, no medication has been proven to cure GAVE.

GAVE is a young disease, with no clear documentation in ancient texts. Traditional Chinese medicine follows a holistic approach based on syndrome differentiation and treatment. By considering the overall manifestations of people, specific herbal medications can be chosen for GAVE. Finding effective herbal treatments and isolating key active compounds could potentially manage GAVE.

## 4. Conclusions

As a rare disease, through this delayed case, much can be gleaned about GAVE. Its diagnosis needs a further clarification. And there is significant room for improvement in treatment strategies. Maybe a combination of endoscopic and medication treatment will be a better choice to GAVE. With the developments of new technologies and methods, the understanding of GAVE will be more comprehensive.

## Author contributions

**Conceptualization:** Zheke Fang, Liangjun Yang.

**Data curation:** Zheng Fang, Qiang Hu.

**Formal analysis:** Zheng Fang, Qiang Hu.

**Writing – original draft:** Zheke Fang, Jiajie Zhu.

**Writing – review & editing:** Zheke Fang, Liangjun Yang.
